# Vectorial Diffractive Neural Metasurfaces for Multiplexed Synthesis of Optical Skyrmions

**DOI:** 10.1002/nap2.70276

**Published:** 2026-08-28

**Authors:** Tianqu Chen, Xu Xing, Mingfeng Xu, Yong Yu, Xi Tang, Yinghui Guo, Fei Zhang, Mingbo Pu, Xiangang Luo

**Affiliations:** ^1^ State Key Laboratory of Optical Field Manipulation Science and Technology Institute of Optics and Electronics Chinese Academy of Sciences Chengdu China; ^2^ Research Center on Vector Optical Fields Institute of Optics and Electronics Chinese Academy of Sciences Chengdu China; ^3^ College of Materials Science and Opto‐Electronic Technology University of Chinese Academy of Sciences Beijing China; ^4^ Sichuan Provincial Engineering Research Center of Digital Materials Chengdu China

**Keywords:** bimeron, inverse design, metasurface, optical skyrmions, VDNN

## Abstract

The synthesis of complex vectorial light fields with nontrivial topological polarization textures requires the coordinated control of spatial amplitude, phase, and polarization. A central challenge is to realize such multidimensional control in a compact physical architecture while retaining multiplexing capability. Here, we propose a vectorial diffractive neural network (VDNN) based on three cascaded metasurfaces for compact passive synthesis of topological vector light fields. By jointly encoding phase and polarization responses across the cascaded layers, the VDNN enables spatially multiplexed generation of multiple skyrmionic and bimeronic states. In a representative design, we demonstrate up to 16 topological textures with numerically evaluated average skyrmion numbers of |s|>0.86, and an average orbital angular momentum (OAM) mode purity exceeding 90%. Full‐wave simulations and tolerance analysis further show that this functionality can be preserved under realistic meta‐atom responses and inter‐layer perturbations. This work provides an inverse‐design approach for compact and multiplexed synthesis of complex topological vector light fields for potential applications in free‐space optical communications, particle manipulation, and polarization imaging.

## Graphical abstract

A three‐layer Jones‐matrix‐based vectorial diffractive neural network enables compact, passive, and multiplexed synthesis of optical skyrmions and bimerons. By mapping distinct Gaussian‐beam input channels to coaxial orbital‐angular‐momentum and polarization states, the device generates up to 16 topological vector textures with high mode purity, robust topological characteristics, and broad potential for integrated multidimensional photonic systems.

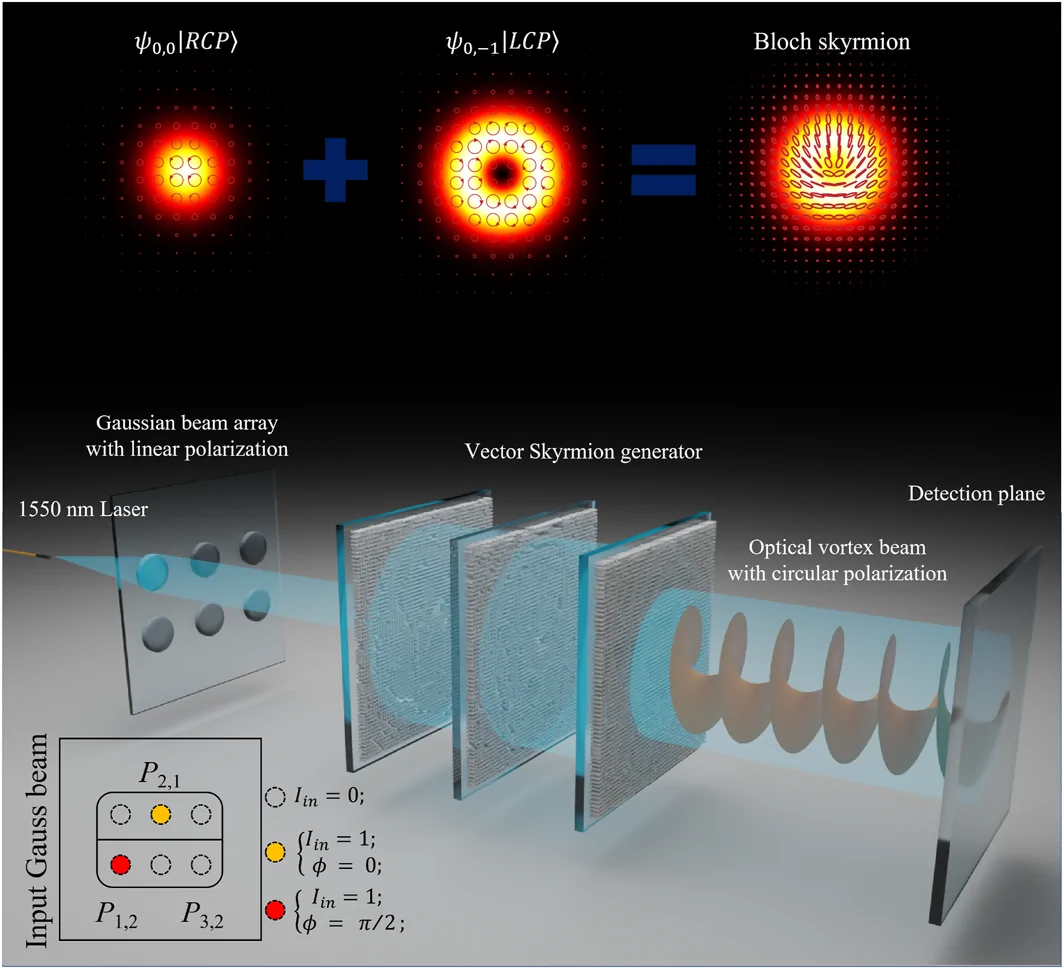

## 1 Introduction

Multidimensional optical field manipulation has become a focal point in optical communications and an important research direction in diffractive neural networks (DNNs) [1, 2, 3, 4, 5]. Vector optical fields feature spatially varying amplitude and polarization [6, 7], with their polarization states mappable onto the Poincaré sphere [8], forming various topological structures which offer new degrees of freedom for applications, such as optical trapping, quantum communication, and polarization‐resolved imaging [9, 10, 11]. Traditional approaches for complex vector optical field manipulation include single‐layer metasurfaces and digital holography. Metasurfaces, as two‐dimensional optical devices with sub‐wavelength scale optical field control capabilities, can effectively modulate the polarization state of optical fields [12, 13, 14]. Moreover, digital holography has enabled dynamic transitions among vectorial textures, revealing their generation and annihilation mechanisms [15, 16, 17]. However, the control degrees of freedom offered by single‐layer metasurfaces are insufficient for high‐capacity multiplexing and high‐precision field modulation [18], whereas current manipulation platforms of digital holography still rely on discrete components, such as SLMs, waveplates, and polarizers, and can generate a limited number of vectorial optical fields, leading to complex setups that hinder integration [17, 19].

Conventional scalar DNNs based on cascaded diffractive layers have been introduced to realize multi‐channel, low‐crosstalk, low‐loss, and highly integrated mode‐manipulation devices [20, 21]. Numerous previous studies focused on classification, recognition or generating and multiplexing scalar optical‐field modes using SLMs and geometric‐phase elements [22, 23, 24, 25, 26, 27], whereas several studies have demonstrated the capability of scalar diffractive neural networks or metasurfaces to generate and manipulate vectorial optical fields [28, 29, 30]. However, the inverse design and multiplexed synthesis of diverse topological vectorial textures using Jones‐matrix‐based vectorial diffractive neural networks remain underexplored (Supporting Information S1: Note S1). This inherently deprives previous studies of one manipulation degree of freedom and a vast number of control channels. To impart a DNN with genuine polarization‐control capabilities, each layer should be implemented as a metasurface to apply an arbitrary, locally varying Jones matrix rather than a single scalar phase delay [22, 31, 32].

Here, we propose a novel vector optical field manipulation architecture called vectorial diffractive neural network (VDNN) based on three‐layer cascaded metasurface. Taking skyrmions, a class of topological textures in vector light fields, as an example, we realize multichannel multiplexing and fine control of vector field patterns for up to 16 skyrmions and bimerons, including Néel, Bloch, and anti‐type, using only three metasurface layers, each comprising merely 60 × 60 meta‐atoms. Invariance of the skyrmion number attests to their topological protection, and all maintain an absolute overlap value above 0.82. The simulation results are in good agreement with the theoretical results. The proposed VDNN architecture successfully balances high integration with a large degree of multiplexing freedom, offering a practical route toward highly integrated photonic applications.

## 2 Theory

### 2.1 Optical Skyrmion

Vector optical fields form topological structures such as skyrmions [33], bimerons [34], and hopfions [35]. Among them, optical skyrmions, characterized by topological protection and tunable polarization textures, have emerged as a central research focus [36]. Originating in high‐energy and condensed‐matter physics [37, 38, 39], skyrmions are three‐dimensional vector‐field configurations localized in space [40], commonly classified as Néel‐type, Bloch‐type, and anti‐skyrmions [15, 41, 42]. As an optical topological quasiparticle represented by Stokes vectors, optical skyrmions are quantitatively characterized by the topological invariant s, which is called the skyrmion number and expressed as follows [37, 43]:

(1) $$s=\frac{1}{4\pi }\underset{\sigma }{\iint }n\cdot \left(\frac{\partial n}{\partial x}\times \frac{\partial n}{\partial y}\right)dxdy$$

where n(x,y) is a three‐dimensional unit‐vector field defined on the two‐dimensional plane σ. For a Stokes‐type skyrmion, the vector field n(x,y) at each spatial position corresponds to a point on the Stokes sphere, whereas the aggregate of these points constitutes a topological mapping from the two‐dimensional plane to the sphere [44]. The skyrmion number s quantifies the number of times the Stokes sphere is fully “wrapped” by this mapping.

Optical skyrmions can be generated by superposing a pair of Laguerre–Gaussian (LG) modes with orthogonal polarizations (Supporting Information S1: Note S2). This process can be expressed as follows [15, 45]:

(2) $$\Psi =\psi_{1}R+\psi_{2}L$$

where Ψ denotes the eigenstate of an optical skyrmion, $\psi_{1}$ and $\psi_{2}$ are the pair of LG modes, R and L represent the right‐ and left circular polarization (RCP and LCP) basis vectors, respectively. In this work, the fundamental Gaussian mode and low‐order Laguerre–Gaussian (LG) modes are adopted as $\psi_{1}$ and $\psi_{2}$.

(3) $$\Psi =\psi_{0,0}R+\left[cos\theta e^{-i\phi }\psi_{\rho ,l}+sin\theta e^{i\phi }\psi_{\rho ,-l}\right]L$$

where $\psi_{0,0}$ is the fundamental Gaussian mode, and $\psi_{\rho ,l}$ denotes an LG mode, where ρ governs the radial multi‐twist structure of the skyrmion and l controls the internal vorticity which is also the skyrmion order. The angles ϕ and θ correspond, respectively, to the azimuthal angle and the polar angle of the eigenstate on the sphere. As a proof of concept, this work exclusively considers first‐order skyrmions, where ρ=0 and |l|≤1, employing six OAM beams whose topological charges are chosen from the set {−1, 0, 1}.

### 2.2 Diffractive Neural Network (DNN) Design

DNNs establish mappings between input and output optical fields by precisely controlling the diffraction and propagation of light [20]. The forward propagation process is modeled using the angular spectrum method [46, 47]. Unlike the recognition and classification tasks undertaken by typical optical diffractive neural networks, this work focuses on optimizing the design of the architecture for precise multidimensional optical field multiplexing and manipulation. Therefore, the loss function used for gradient‐based optimization through backpropagation is designed to emphasize the optimization of optical field manipulation performance. This work mainly employs the cross‐correlation function, which can quantify the similarity of complex‐valued electric fields, and the mean squared error function, which can evaluate deviations in the energy distribution, as loss functions.

(4) $$\begin{array}{cc} L_{\mathrm{corr}} & =1-\frac{|\iint E_{\mathrm{out}}\cdot E_{\mathrm{tar}}^{*}dxdy{|}^{2}}{\iint |E_{\mathrm{out}}{|}^{2}dxdy\cdot \iint |E_{\mathrm{tar}}{|}^{2}dxdy}\\ L_{\mathrm{MSE}} & =\frac{\iint {\left(|E_{\mathrm{out}}{|}^{2}-|E_{\mathrm{tar}}{|}^{2}\right)}^{2}dxdy}{\iint dxdy} \end{array}$$

where the loss function based on cross‐correlation is denoted by $L_{\mathrm{corr}}$. $E_{\mathrm{tar}}\left(x,y\right)$ is the target electric field distribution. A value closer to zero, the more correlated the two fields are. $L_{\mathrm{MSE}}$ represents the mean squared error (MSE) loss function based on light intensity distribution. $|E_{\mathrm{out}}\left(x,y\right){|}^{2}$ and $|E_{\mathrm{tar}}\left(x,y\right){|}^{2}$ represent the intensity values of the output field and the target field at the (x,y) position, respectively. The total loss $L_{\mathrm{tot}}$ is obtained by taking the weighted average of the two loss functions to precisely control the phase and intensity distribution of the output optical field.

In addition, to the degrees of freedom associated with scalar optical fields, polarization represents an important dimension in optical systems and plays a central role in the VDNN [48, 49]. To achieve full polarization control of the vector optical field, it is necessary to combine Jones matrix and gradient backpropagation. The resulting numerical operations remain differentiable, enabling integration into DNN training (Supporting Information S1: Note S3) [50].

As shown in Figure 1, this work realizes mode conversion and beam combining from a Gaussian array to a skyrmion. Therefore, the input layer receives Gaussian beams at different positions, representing different OAM components. These beams are decomposed into their x‐ and y‐polarized components. The output layer produces the corresponding coaxial OAM component combinations as skyrmions. During the training process, the Adam optimizer was utilized to train the parameters in order to enhance convergence.

**FIGURE 1:**
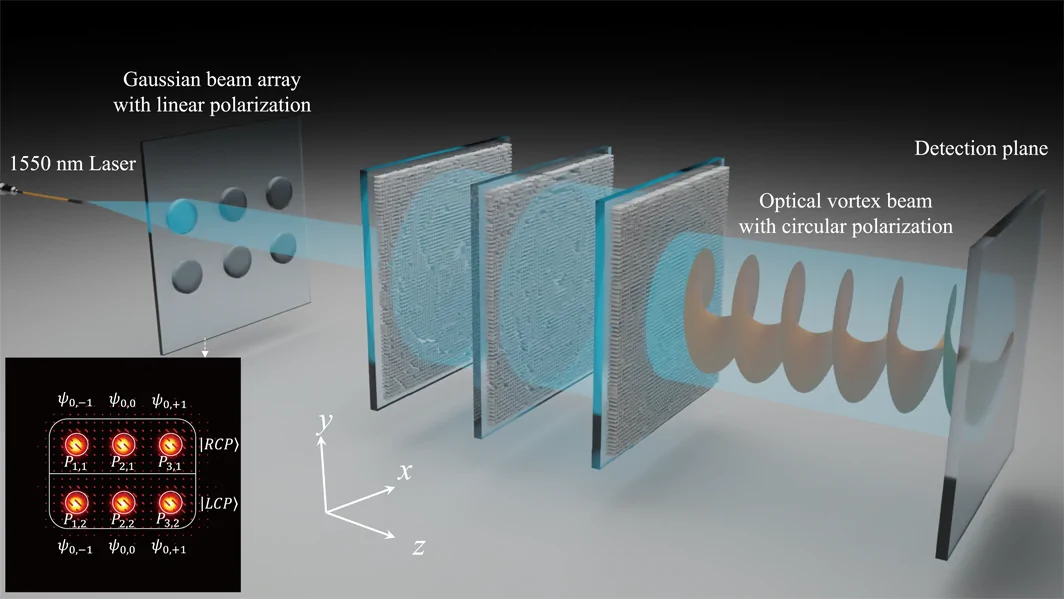
System architecture of VDNN with three‐layer metasurface. Gaussian beams from different positions of the 2 × 3 fiber array, all with 45° linear polarization (as illustrated in the inset), are incident on the cascaded metasurfaces and are converted into coaxial circularly polarized OAM beams. The Stokes‐type skyrmion textures synthesized by beam combining can be obtained on the detection plane.

### 2.3 Design on Metasurface

This work integrates the Jones‐matrix‐based method of metasurfaces into the training process of VDNN. The optical‐field distributions of the training set are obtained by decomposing the input and output vector fields onto an orthogonal polarization basis. The trainable variables are grouped as a parameter group $\gamma \in \left\{\phi_{x},\phi_{y},\theta \right\}$ (phase delays along the fast and slow axes $\phi_{x}$ and $\phi_{y}$, rotation angle θ) into the parameter training part of the neural network. Finally, the optimal values of the parameters are obtained through gradient backpropagation, achieving full polarization control of the vector optical field. The parameters are then selected from a library of meta‐atoms [51]. To ensure the overall efficiency of the device, the transmission coefficient of the meta‐atoms denoted by the amplitude modulation parameter $t^{k}\left(x,y\right)$ is fixed at unity during the training process. Although under specific requirements, amplitude modulation can also be conveniently incorporated as a trainable parameter and an additional degree of design freedom in the VDNN optimization process.

## 3 Result and Discussion

A vectorial optical field training set is obtained by compensating for the propagation distance and constraining the optical‐field energy. This approach effectively avoids convergence issues arising from the divergent behavior of OAM beam propagation and overall optical field energy mismatches (the detailed simulation design process, and the architectural parameters in detail are shown in Supporting Information S1: Notes S4 and S5). Figure 2a shows the final distribution of training parameters for the vector diffraction neural network, in which the parameters of meta‐atoms have the following ranges: $\phi_{x},\phi_{y}\in \left[0,2\pi \right]$, θ∈[0,π].

**FIGURE 2:**
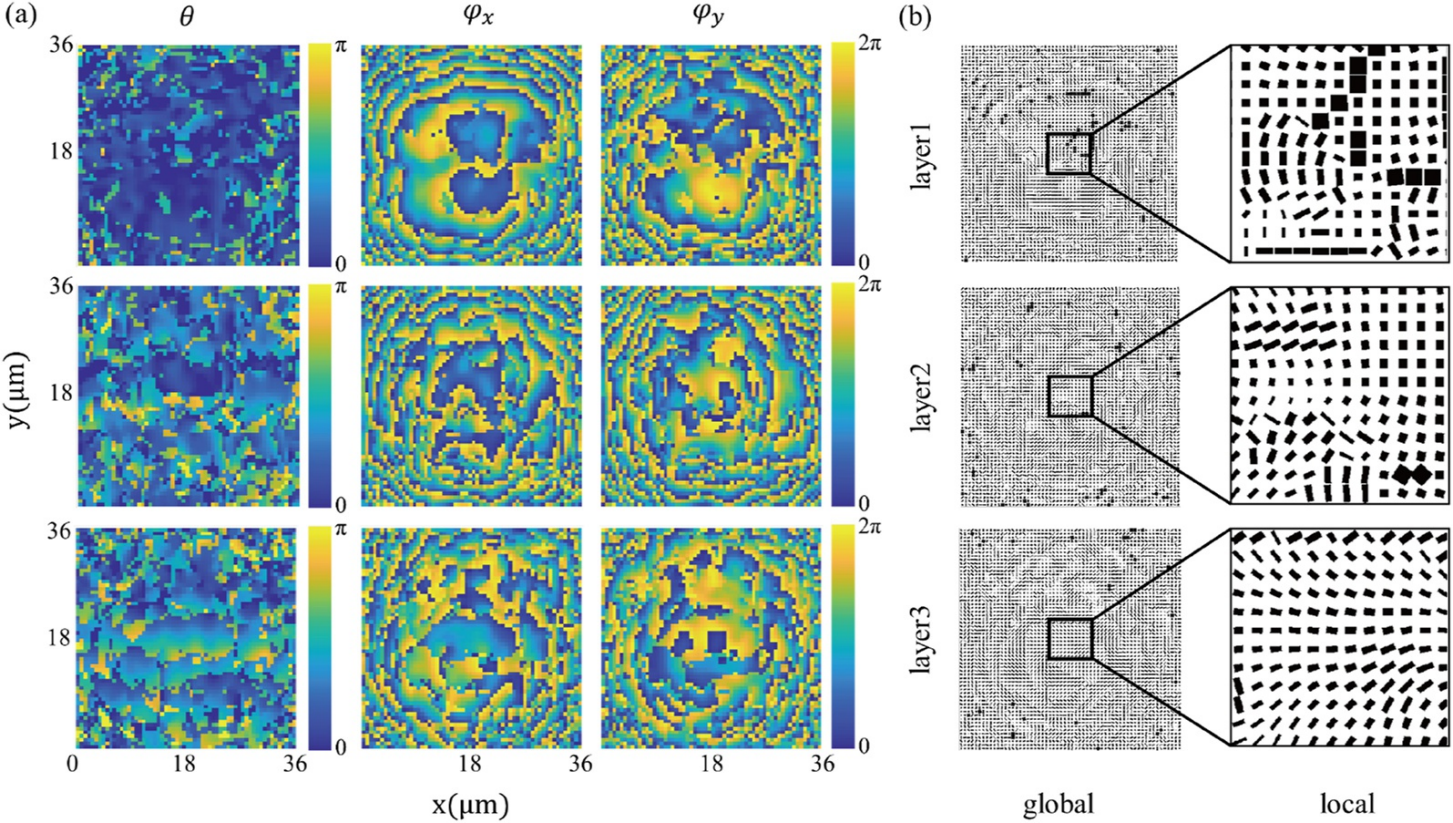
(a) Training results of the Jones matrix parameters for the three‐layer metasurfaces in VDNN. (b) Layout diagram of the three‐layer metasurface structure and its enlarged local view.

According to the trained Jones matrix distribution, the most suitable meta‐atoms are selected from the pre‐prepared meta‐atom unit library. Figure 2b shows the meta‐atom distribution of the VDNN. When selecting the meta‐atoms, the phase distribution of the entire metasurface can be matched in priority by reducing the transmittance of some meta‐atoms. It can be seen that the transmittance of a few meta‐atoms drop to around 50%, but overall transmittance still remains at a high level (Supporting Information S1: Note S7). The average transmittance T of each layer for x and y polarizations is approximately 0.92.

To verify the function of our VDNN, we employ both numerical simulation and finite‐difference time‐domain (FDTD) simulations using Lumerical FDTD. Figure 3 displays the optical‐field distributions under different incident Gaussian beams sharing the same 45° linear polarization for both methods, with energy conversion efficiency annotated. It can be seen that both methods can efficiently control the target OAM and spin angular momentum modes, with overall optical field distribution closely resembling the ideal one. Aside from difference in conversion efficiency, there is little distinction between the two methods in terms of optical field morphology. Therefore, subsequent sections only present texture results generated by FDTD, whereas in the following text focusing on performance analysis, the differences between two methods will be taken into account.

**FIGURE 3:**
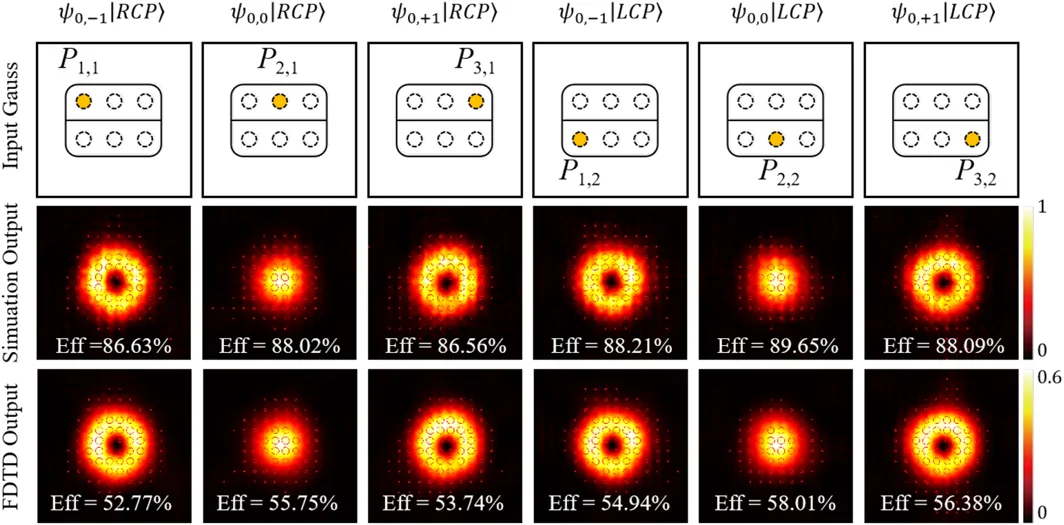
Comparison of the output intensity distributions, polarization textures, and optical energy conversion efficiencies obtained from numerical and FDTD simulations for different input Gaussian‐beam positions sharing the same 45° linear polarization.

To further illustrate the physical transformation process in the proposed VDNN, Figure 4 presents the field evolution under several representative Gaussian‐array excitations. As shown in the upper panel, the incident field is successively processed by three cascaded metasurface layers before forming the final output distribution. The lower panel demonstrates that different input‐channel combinations lead to distinct intermediate field distributions and output patterns. For each excitation case, the optical field is gradually reshaped as it propagates through the cascaded structure, indicating that the required spatial redistribution is not achieved by a single abrupt modulation, but rather by a layer‐by‐layer cooperative transformation. This result highlights the physical mechanism of the cascaded architecture, in which multiple diffractive layers jointly implement the mapping from the input Gaussian‐array channels to the desired target fields while maintaining relatively high conversion efficiency.

**FIGURE 4:**
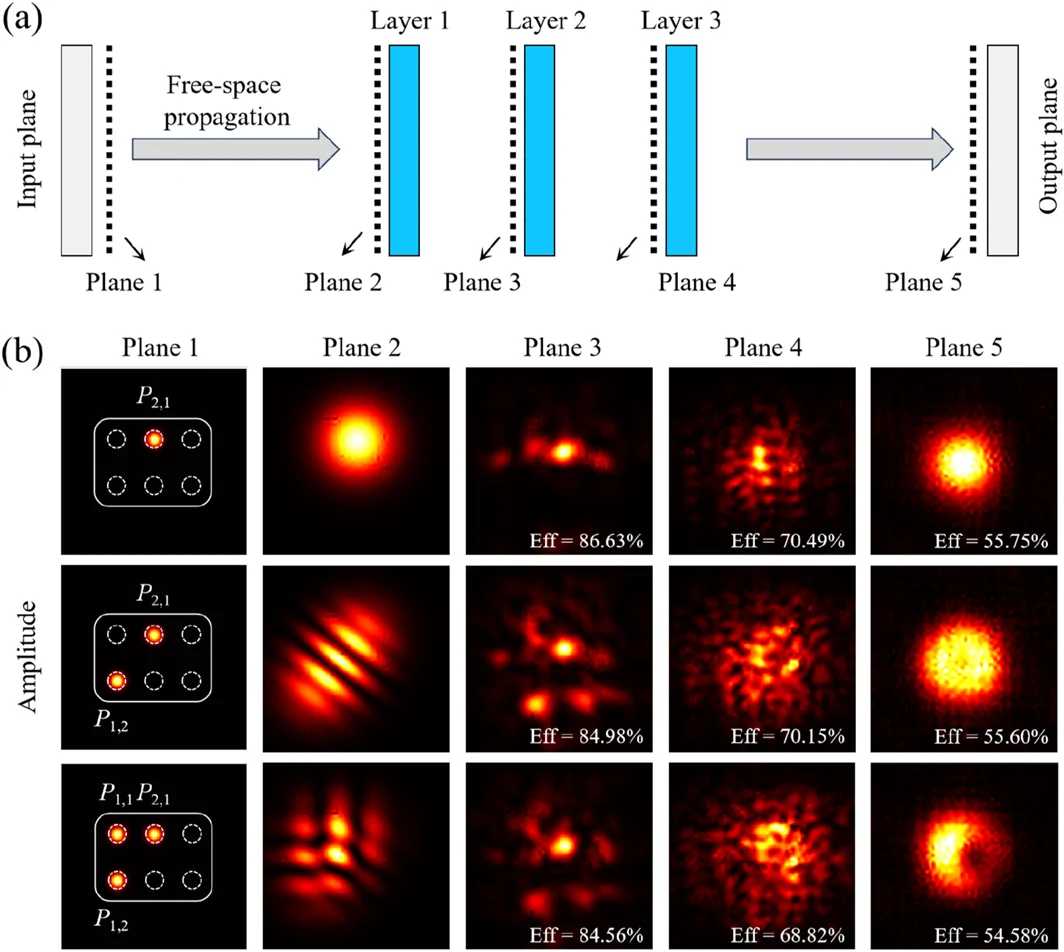
(a) Field transformation process of the cascaded three‐layer metasurface under different Gaussian‐array excitations. (b) Incident field distributions for three representative cases and the corresponding field evolution with associated conversion efficiencies.

Then, the design performance of the three‐layer inverse‐designed metasurface‐based VDNN, through full‐wave FDTD simulations, has been shown in Figure 5. The figure displays the intensity distribution pattern of the incident Gaussian beam array, the distributions of the output optical field, polarization state (locally enlarged), and Stokes vector field. As shown in Figure 5a, in the case of illumination at positions $P_{1,2}$ and $P_{2,1}$, and with a phase difference of 0 between the two Gaussian beams, the output beam conforms to the distribution characteristics of the Néel‐type Stokes skyrmion. As can be seen from the locally enlarged polarization state distribution, the center is dominated by the right‐handed component, while the edges are dominated by the left‐handed component. Moreover, the hedgehog‐like texture structure is evident in the Stokes vector field distribution, which is consistent with the typical topological texture of the Néel type. The calculated skyrmion number is around s=0.879, which matches well with the target field value.

**FIGURE 5:**
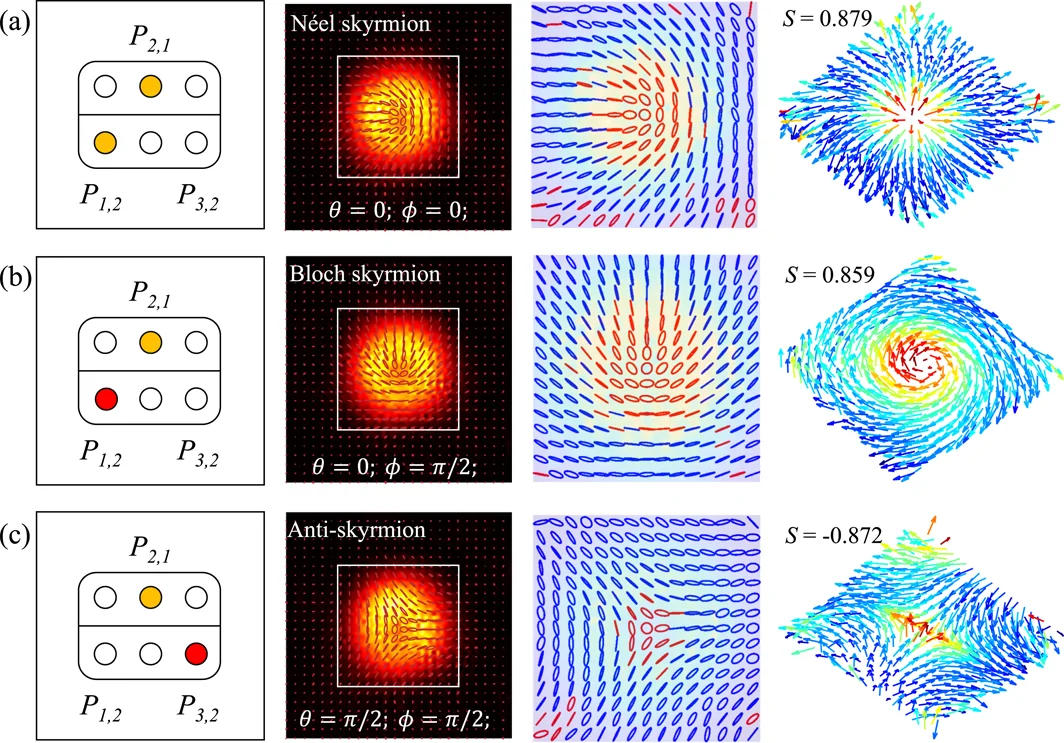
Distributions of Gaussian beam array intensity, output optical field, polarization state (locally enlarged), and Stokes vector field of (a) Néel skyrmion. (b) Bloch skyrmion. (c) Anti‐skyrmion. The different colors of the incident Gaussian spots in the first column indicate a phase difference of $\frac{\pi }{2}$ between the two fundamental Gaussian beams. In the third column, the colors of the ellipses represent polarization handedness, with red indicating right‐handed polarization and blue indicating left‐handed polarization.

Furthermore, Figure 5b shows the Bloch‐type Stokes skyrmion generation case. Compared with the ideal Bloch‐type skyrmion, the vector field distribution features a high degree of consistency, and it has a distinct spiral‐like topological texture. Additionally, the skyrmion number is s=0.859, which also matches well with the target field value. The slight decrease in the skyrmion number may come from non‐global optimal solution, transmission loss and local periodic approximation (Supporting Information S1: Note S8).

By illuminating $P_{3,2}$ and $P_{2,1}$, as shown in Figure 5c, the output polarization vectors can be obtained, forming a non‐radial or rotationally asymmetric optical field distribution with a saddle‐like topological texture, where the vectors converge from different directions in the four quadrants. The skyrmion number s=−0.872 indicates that its topological structure is opposite in direction to that of the standard skyrmion (s>0). These characteristics indicate that it is an anti‐type skyrmion (The characterization of remaining five skyrmion states is specifically listed in Supporting Information S1: Note S11). Overall, the design of VDNN supports the high‐quality generation and rapid modulation of eight types of skyrmions.

Apart from the common types of skyrmions mentioned above, the VDNN structure can also conveniently generate and modulate various bimerons. A bimeron is a type of vector optical field structure composed of two topological excitations, with its vector field having two mapping paths from the south pole to the north pole in space, resulting in a double‐centered topological structure. The synthesis method is similar to that of a Stokes‐type skyrmion, and it can be achieved by replacing the LCP component in Equation (3) with a linear horizontally polarized component. The field synthesis can be expressed as follows [34]:

(5) $$\Psi =\psi_{0,0}R+\left[cos\theta e^{-i\phi }\psi_{\rho ,l}+sin\theta e^{i\phi }\psi_{\rho ,-l}\right]H$$

where H denotes the horizontally polarized basis vector. As an optical quasi‐particle with the same topological texture as skyrmions, bimerons and skyrmions can be converted into each other.

Here, a horizontally polarized −1 order OAM beam can be generated at the output by superposing left‐ and right‐circularly polarized OAM beams, both with a topological charge of −1. Analogous to Equation (5), as shown in Figure 6a,b, the control of the phase difference between the horizontally polarized OAM beam and the fundamental Gaussian beam can be used to regulate Bloch‐type and Néel‐type bimerons. Similarly, by changing the incident Gaussian beams, a horizontally polarized order +1 OAM beam can be obtained. Finally, an anti‐type bimeron can be generated, as shown in Figure 6c. The skyrmion numbers of the three bimerons are shown in the figure, which shows the overall match is good (the other five types of bimerons are shown in Supporting Information S1: Note S12).

**FIGURE 6:**
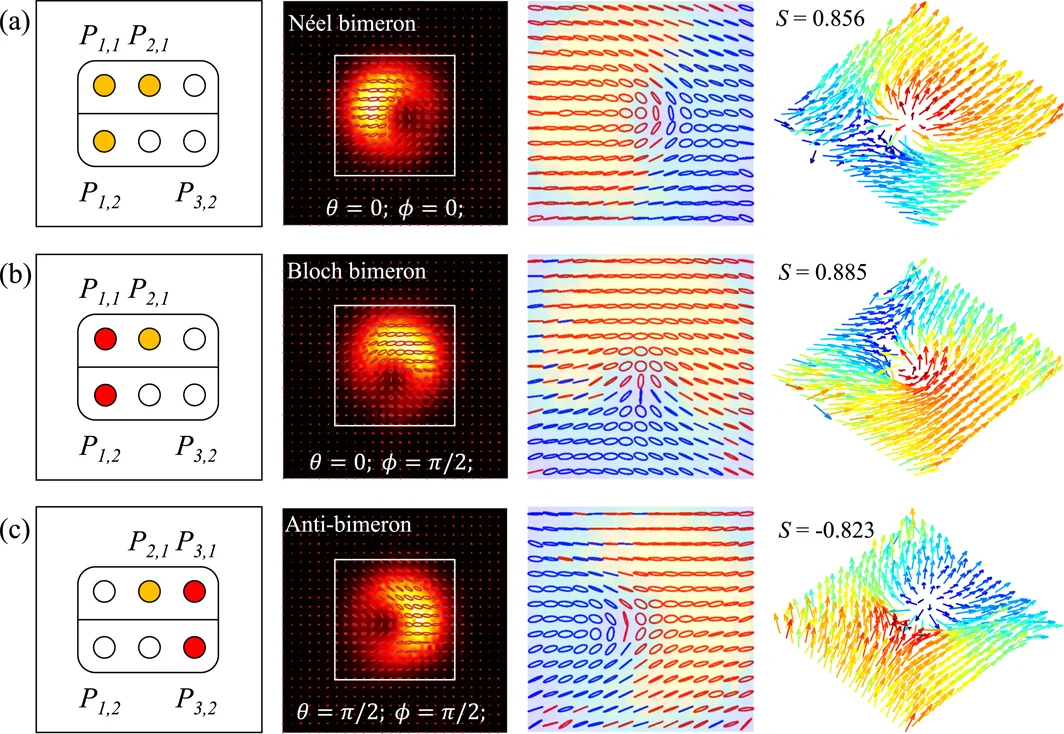
Distributions of Gaussian beam array intensity, output optical field, polarization state, and Stokes vector field of (a) Néel bimeron. (b) Bloch bimeron. (c) Anti‐bimeron. In the fourth column, two completely vertical arrows indicate the center of the bimeron.

To investigate the impact of transmittance spectrum on device performance, the amplitude and phase distributions and topological purity matrix are obtained from numerical simulation (without transmission loss) and FDTD simulation (with transmission loss). As shown in Figure 3, the impact of meta‐atom transmittance deviation is a global decrease in energy conversion efficiency and has a relatively small impact on the optical field pattern and phase distribution. As for mode purity shown in Supporting Information S1: Figure S11, numerical values for all modes cluster around 0.9, regardless of whether transmission loss is considered. This phenomenon is consistent with amplitude and phase distribution of the output optical field, so that the impact of transmission loss on the optical field mainly causes overall decrease in conversion efficiency, with limited influence on optical field quality (Supporting Information S1: Note S13).

Alignment errors may occur during the actual assembly and adjustment of the three‐layer metasurface. Therefore, it is necessary to evaluate the impact of alignment errors on the purity of OAM and the overall optical field conversion efficiency. The alignment errors can be categorized into the lateral, longitudinal displacement and rotational errors of the metasurface ($X_{\mathrm{offset}}$, $Y_{\mathrm{offset}},\theta$) and the layer spacing error $\left(D_{\mathrm{offset}}\right)$ (Supporting Information S1: Note S14). Lateral and longitudinal displacement errors should focus on the later diffraction layers, and the errors should be controlled within two pixels as much as possible. If the layer spacing error is controlled within the range [−2λ,2λ], its impact on OAM mode purity and overall optical field conversion efficiency is relatively small. Overall, the impact of layer spacing errors on device performance is much smaller than that of lateral and longitudinal alignment errors (Supporting Information S1: Figure S16). Therefore, in subsequent practical experiments, lateral and longitudinal errors should be the primary focus.

## 4 Conclusion

This work demonstrates the VDNN‐based generation and modulation of high‐quality Stokes skyrmions comprising multiple optical modes. The proposed system employs a three‐layer cascaded metasurface architecture and supports the generation and switching of up to 16 representative first‐order Stokes skyrmion and bimeron states. It delivers exceptional control precision and vector‐field fidelity, exhibiting excellent agreement and stability with FDTD simulations in all key observables, including the output polarization texture, vector distribution, and topological charge. The system achieves an average optical‐field conversion efficiency of η≈55%, an average skyrmion number |s|>0.86, and an average OAM‐mode purity Γ>90%. Compared with digital holography, the proposed architecture leverages inverse design to integrate phase modulation, polarization control, and beam combination into a single, passive device. Because no dynamic reconfiguration of the metasurface itself is required, the system is not limited by the refresh rate of the modulation device and therefore has the potential to support rapid input‐channel switching.

This work validates the feasibility of combining VDNN inverse design with cascaded metasurface implementation and demonstrates the potential of this approach for the precise manipulation of vectorial optical fields. These results provide a foundation for future applications in optical communications, optical computing, and optical data storage.

## Author Contributions

**Tianqu Chen:** software, formal analysis, writing – original draft, investigation, methodology, validation. **Xu Xing:** software, investigation, validation, formal analysis, writing – original draft, visualization. **Mingfeng Xu:** conceptualization, methodology, supervision, funding acquisition, writing – review and editing, project administration, visualization. **Yong Yu:** formal analysis, validation, visualization. **Xi Tang:** validation. **Yinghui Guo:** formal analysis. **Fei Zhang:** formal analysis. **Mingbo Pu:** writing – review and editing, resources, methodology. **Xiangang Luo:** writing – review and editing, conceptualization, project administration, resources, methodology.

## Funding

This work is supported by the National Natural Science Foundation of China (NSFC) (Grants 62575286 and U24A6010) and the Sichuan Science and Technology Program (Grant 2026NSFSCZY0127).

## Conflicts of Interest

The authors declare no conflicts of interest.

## Supporting information


Supporting Information S1

## Data Availability

The data that support the findings of this work are available from the corresponding author upon reasonable request.
