# Supplementary material for: Vectorial Diffractive Neural Metasurfaces for Multiplexed Synthesis of Optical Skyrmions
Source: Nanophotonics. 2026 Aug 28;15(17):e70276. doi: 10.1002/nap2.70276 (PMC13672138; doi:10.1002/nap2.70276)
Supplement: Supplementary file 1 [file NAP2-15-e70276-s001.docx]

Supporting Information

**Vectorial Diffractive Neural Metasurfaces for Multiplexed Synthesis of Optical Skyrmions**

*Tianqu Chen****^†^****, Xu Xing****^†^****, Mingfeng Xu, Yong Yu, Xi Tang, Yinghui Guo, Fei Zhang, Mingbo Pu*, Xiangang Luo**

State Key Laboratory of Optical Field Manipulation Science and Technology, Institute of Optics and Electronics, Chinese Academy of Sciences, Chengdu, 610209, China

Research Center on Vector Optical Fields, Institute of Optics and Electronics, Chinese Academy of Sciences, Chengdu, 610209, China

College of Materials Science and Opto-Electronic Technology, University of Chinese Academy of Sciences, Beijing, 100049, China

Sichuan Provincial Engineering Research Center of Digital Materials, Chengdu, 610209, China

E-mail: pmb@ioe.ac.cn, lxg@ioe.ac.cn

Note S1. Comparison of vectorial optical-field manipulation systems

Table S1 summarizes representative previous studies on the generation and manipulation of complex vectorial optical fields. SLM- and DMD-based systems offer dynamic reconfigurability but generally require bulky external optical setups, whereas metasurface-based systems provide greater compactness and integration at the expense of passive, predesigned operation. Meanwhile, recent inverse-design approaches have markedly increased multiplexing capacity. Combined with metasurfaces, they enable higher channel counts despite limited device reconfigurability. Although single-layer metasurfaces are easier to fabricate and align, cascaded architectures provide greater modulation freedom at the cost of increased fabrication and alignment complexity. The present work employs the VDNN architecture and inverse-design strategy to realize the generation, multiplexing, and manipulation of complex optical-skyrmion textures.

Table S1. Summary of vector optical field manipulation systems.

Int.: Integration; Rec.: Reconfigurable; Exp.: Experimental; Simu.: Simulation

| Ref. | System | Int. | Output | Capacity | Validation | λ (nm) | Algorithm | Rec. |
| --- | --- | --- | --- | --- | --- | --- | --- | --- |
| [1] Yue, et al.,2016 | Single Metasurface | Yes | Vectorial optical mode | 2 | Exp. | 697 | Forward design | No |
| [2] Shen, et al.,2022 | SLM | No | Skyrmions | >8 | Exp. | 632.8 | Forward design | Yes |
| [3] Singh, et al.,2023 | DMD | No | Skyrmions | >3 | Exp. | 632.5 | Forward design | Yes |
| [4] Teng, et al.,2023 | Dual-SLM | No | Skyrmions | >16 | Exp. | 633 | Forward design | Yes |
| [5] Wang, et al.,2024 | Single Metasurface | Yes | Holographic | 55 | Exp. | 3000 | Inverse design  (VDNN) | No |
| [6] Soma, et al.,2025 | Cascaded Metasurface | Yes | Vectorial optical mode | 6 | Exp. | 1550 | Inverse design  (Adjoint method) | No |
| [7] Zhen, et al.,2026 | SLM | No | Skyrmions | >12 | Exp. | 532 | Forward design | Yes |
| [8] Hu, et al.,2026 | SLM | No | Vectorial optical mode | 16 | Exp. | 633 | Inverse design  (DNN) | Yes |
| [9] Xiao, et al.,2026 | SLM | No | Vectorial optical mode | 48 | Exp. | 633 | Inverse design  (DNN) | Yes |
| **This work** | **Cascaded Metasurface** | **Yes** | **Skyrmions** | **16** | **Simu.** | **1550** | **Inverse design**  **(VDNN)** | **No** |

Note S2. Generation and graphical representation of skyrmions

In Figure S1, the normalized Stokes vector components $(S_{1}/S_{0},S_{2}/S_{0},S_{3}/S_{0})$ are mapped onto unit vectors in Cartesian coordinates $(x,y,z)$, where the arrow orientation indicates the direction of the Stokes vector and its z-component encode the spin angular-momentum chirality with the sign of $S_{3}$ (right-handed / left-handed). Color encodes the degree of circular polarization, corresponding to $S_{3}/S_{0}$. The overall Bloch-type Stokes vector field exhibits a spiral topological texture. In contrast, the Néel and antiskyrmion textures manifest hedgehog-like and saddle-like patterns, respectively. Figure S1(b) shows that optical skyrmions can be generated by superposing a pair of Laguerre–Gaussian (LG) modes with orthogonal polarizations.

Figure S1. (a) Stokes-vector-field distribution and topological charge of Bloch-type skyrmion. (b) Generation of ideal Bloch-type skyrmions via co-axially superposed optical fields

Note S3. Backward propagation of VDNN

To achieve the optimal design of each phase control element in the optical field manipulation network, it is necessary to utilize the back propagation algorithm to solve the gradient of the loss function, thereby iteratively optimizing the network parameters. Since this work involves pure phase modulation, the key is to calculate the gradient of with respect to the phase variables in the phase modulation matrix $\Phi^{k}$. By the chain rule for partial derivatives of complex functions, we obtain:

Here, $N$ is the output layer, $\dagger$ denotes the conjugate transpose, and expanding recursively layer by layer, we obtain:

Because $U=F^{-1}HF\left[\cdot\right]$is an operator, we have:

Substituting, we obtain the final result:

Forward propagation is achieved via a series of operators $U$ and phase modulation matrices $\Phi^{l}$. Taking the conjugate of the path product $\prod_{l=k+1}^{N} U_{l}\Phi^{l}$gives $\left(\prod_{l=k+1}^{N} U_{l}\Phi^{l}\right)^{\dagger}$, which physically represents backward optical field propagation.represents the gradient direction propagation path.

Besides dimensions of scalar field, polarization, as an important dimension in optical systems, plays a significant role in DNN. For optical fields incident normally on a polarization-modulating device, their polarization state can be represented by two orthogonal basis polarization states, such as LCP & RCP. Their two-dimensional Jones vector is

$$|LCP\rangle=\left[\begin{matrix} 1 \\ i \end{matrix}\right],|RCP\rangle=\left[\begin{matrix} 1 \\ -i \end{matrix}\right]$$

To achieve full polarization control of the vector optical field, it is necessary to provide independent phase modulation effects $\varphi_{1}\left( x,y \right)$ and $\varphi_{2}\left( x,y \right)$ for the two basis vectors. The modulation effects can be represented by a Jones matrix as follows

During the design, propagation- and geometric-phase based metasurfaces are generally assumed to be lossless. Moreover, the polarization transformation preserves orthogonality, implying that the polarization Jones matrix ($J(x,y)$) satisfies both the unitarity and symmetry conditions. Accordingly, the Jones matrix can be represented in the standard eigendecomposition form of a unitary matrix:

$$J\text{(}x,y\text{)=}R\Lambda R^{-1}$$

Here, $R$denotes the orthonormal matrix whose columns are the eigenvectors of the Jones matrix $J(x,y)$. Physically, it can be interpreted as a transformation of the polarization basis. The diagonal matrix $\Lambda$ contains the eigenvalues, whose diagonal entries describe the phase responses of the corresponding eigenpolarization states after modulation by the Jones matrix. Consequently, $\Lambda$characterizes the phase modulation imparted by the system to each eigenstate. The eigenvalues and eigenvectors of the Jones matrix can be obtained by solving the characteristic equation (S9), yielding:

$$J(x,y)=\left[\begin{matrix} V_{1} & V_{2} \end{matrix}\right]\left[\begin{matrix} \lambda_{1} & 0 \\ 0 & \lambda_{2} \end{matrix}\right]\left[\begin{matrix} V_{1} & V_{2} \end{matrix}\right]^{-1}$$

$$=\left[\begin{matrix} \cos\theta& \sin\theta\\ -\sin\theta& \cos\theta\end{matrix}\right]\left[\begin{matrix} \lambda_{1} & 0 \\ 0 & \lambda_{2} \end{matrix}\right]\left[\begin{matrix} \cos\theta& \sin\theta\\ -\sin\theta& \cos\theta\end{matrix}\right]$$

Where and are the two eigenvalues of the Jones matrix, and the corresponding eigenvectors are parameterized by the angle.

In this work, the Jones-matrix-based meta-atom design is incorporated into the training process of the optical diffractive neural network. Specifically, the meta-atom Jones matrix parameters, namely the phase shifts,and the rotation angle$\theta$, are directly treated as trainable variables of the neural network. The optimal values of these three parameters are then obtained through gradient backpropagation, thereby enabling full-polarization manipulation of vector optical fields. Accordingly, the output vector field of the *k*-th layer can be described as:

$$\left[\begin{matrix} E_{x,out}^{k}(x,y) \\ E_{y,out}^{k}(x,y) \end{matrix}\right]=J(x,y)\left[\begin{matrix} E_{x,in}^{k}(x,y) \\ E_{y,in}^{k}(x,y) \end{matrix}\right]$$

Expanding Eq. (9) yields

Eq. () explicitly describes the polarization modulation process of the metasurface acting on the incident optical field. The entire formulation is composed of differentiable algebraic operations, including coordinate transformations, complex exponential functions, and matrix multiplications. Therefore, the polarization modulation process can be naturally embedded into the training framework of the optical diffractive neural network, enabling end-to-end optimization via gradient backpropagation and facilitating the efficient inverse design of the meta-atom parameters.

When high numerical aperture propagation conditions are not involved, the polarization components of the optical field along the propagation direction ($z$-axis) can be neglected. In this case, the optical field is described by two orthogonal polarization basis vectors along the $x$ and $y$ directions, and these two components propagate independently. Therefore, the propagation process of the vector optical field can be expressed as：

$$\left[\begin{matrix} E_{x,out}^{i,k+1} \\ E_{y,out}^{i,k+1} \end{matrix}\right]=J^{k+1}F^{-1}HF\left[\begin{matrix} E_{x,out}^{i,k} \\ E_{y,out}^{i.k} \end{matrix}\right]$$

Similar to the gradient backpropagation procedure employed in conventional scalar diffractive neural networks, the Adam optimizer can be used to compute the gradients of the two orthogonal polarization channels independently and update the corresponding meta-atom parameters. As a result, the vector diffractive neural network is trained in an end-to-end manner by matching the incident polarization field to the target optical field. The optimized meta-atom Jones matrix parameters,are directly obtained as the network outputs.

To maximize the overall transmission efficiency of the device, the amplitude transmission coefficient of each meta-atom is fixed at $t^{k}\left(x,y\right)=1$ throughout the optimization process. Nevertheless, when required for specific applications, the amplitude modulation coefficient can also be incorporated into the optimization as an additional trainable parameter, thereby providing an extra degree of design freedom. Finally, according to the optimized meta-atom parameter distributions predicted by the diffractive neural network, a lookup operation is performed within the meta-atom library to identify the corresponding structural geometries, from which the metasurface layout for each diffractive layer is constructed.

Note S4. Simulation network architecture and training set parameters

As shown in Figure S2, the incident optical field of the DNN in this paper is designed as a 2×3 Gaussian mode array that can be realized using a fiber array, arranged in a rectangular pattern in space. Moreover, the polarization state of each Gaussian mode is specified as linear polarization at a 45° angle. The corresponding output beams are six coaxial OAM beams with LCP and RCP. Compared with traditional skyrmion generation systems, this structure does not require complex beam splitting and combining steps. Instead, it achieves coaxial emission of the output beams simply through normal incidence from the fiber array. Considering that an increase in the number of layers leads to an increase in computational resources, experimental fabrication, and alignment difficulties, this paper sets the number of layers for the six-channel vector diffractive neural network to three.

Figure S2. Input Gaussian beam positions and their corresponding coaxial output OAM beams’ optical field.

The trainable meta-atom structural parameters include the phase delay along the long axis (correlated with $l_{x}$), the phase delay along the short axis (correlated with $l_{y}$), and the rotation angle of the meta-atoms $\theta$. The resolution of each metasurface is set to 60×60, resulting in a total number of trainable degrees of freedom of 60×60×3×3=32,400. During training, the initial parameters are randomly distributed. To satisfy the sampling theorem in the Fourier transform, zero-padding can be applied to the input optical field to expand the computational window and avoid frequency aliasing. The loss function is a weighted average of the cross-correlation function and the mean squared error, with their weights set to approximately 1:1 in this work. These weights may need to be adjusted for different design objectives. Here, they were manually tuned based on empirical training performance, while adaptive weighting strategies could be introduced in future work to further improve the algorithmic performance. The Adam optimizer is used with a learning rate of 10^−3^, and the number of training epochs is set to 1000.

Note S5. The practical input-field generation and modulation scheme analysis

The architectural parameters in detail are shown in Figure S3. The incident optical field is a Gaussian beam array with normal incidence at a wavelength of $1550nm$. The Gaussian spot diameter is designed to be$d_{Gauss}=3.6\mu m$, which corresponds to a Gaussian waist radius of $w_{0}=1.8\mu m$, with a lateral spacing of $7.1\mu m$ and a longitudinal spacing of $8.85\mu m$ in the Gaussian spot array. The distance between the Gaussian beam array and the first metasurface is $D_{input}=20\lambda=31\mu m$, and the distance between the last metasurface and the observation plane is $D_{output}=80\lambda=124\mu m$. The interlayer spacing between the three metasurfaces is also $\Delta D=20\lambda=31\mu m$. The unit cell structure period of the meta-atoms is $600nm$. Therefore, the size of incident and output light fields is $L_{x}\times L_{y}=36\mu m\times36\mu m$.

Figure S3. The architecture of the VDNN and diagram of the determined structural parameters

The present design serves as a proof-of-concept demonstration of the vectorial inverse-design capability and multichannel mapping functionality of the Jones-matrix-based multilayer VDNN. Accordingly, the six Gaussian input channels are assigned identical nominal phases and polarization states to avoid introducing additional complexity into the verification of the VDNN architecture. Here, we outline two possible schemes for practical input-field generation and modulation, including an SLM/CGH-based free-space scheme and a compact multicore-fiber scheme with integrated microlenses.

A micrometer-scale $2\times3$ Gaussian-beam array can be generated from single narrow-linewidth laser using a phase-only spatial light modulator (SLM), an optimization-designed computer-generated hologram (CGH), and a demagnifying optical system. Taking the beam diameter as $w_{0}=1.8\mu m$for which the approximate numerical aperture required for diffraction-limited Gaussian focusing is,

$$\text{NA}\approx\frac{\text{λ}}{\text{π}w_{0}}\approx\frac{1.55}{1.8\text{π}}=0.27$$

which can be readily accessible using commercial near-infrared objectives.

Moreover, the normalized channel spacings are $7.08/1.55=4.57\text{λ}$and$8.85/1.55=5.71\text{λ}$, whereas well-resolved arrays with a spacing of $0.952\text{μm}$, corresponding to approximately $1.16\text{λ}$, have been experimentally generated at 820 nm wavelength using an SLM and an NA = 0.75 objective. These results indicate that the spatial scale adopted here is experimentally achievable using established CGH and demagnifying-imaging techniques. ^[^**^10^**^]^

Because all beamlets are derived from the same incident laser field and generated by a common hologram, they share a common coherence reference, while a common polarizer and wave plate can define their incident polarization state. The generated array can subsequently be demagnified and projected onto the first metasurface through relay-imaging optics. In addition to generating the spatial intensity distribution, phase-only CGH methods can provide simultaneous control over the amplitude and phase of the output optical field. Therefore, the positions, relative amplitudes, activation states, and nominal relative phases of the six beamlets can be encoded in the holographic phase pattern. ^[11, 12]^ Switching the pattern displayed on the SLM allows the input-channel combination and phase configuration to be updated dynamically. Independent phase control of coherent beam arrays using an SLM has also been experimentally employed for beam switching, splitting, and focus shaping. Static phase offsets and optical-system aberrations can be calibrated and compensated by incorporating the corresponding correction into the hologram.

Compared with the SLM/CGH and demagnifying relay-imaging scheme, a multicore-fiber-based configuration may provide a more compact alternative. Previous experiments have demonstrated single-mode multicore fibers with a mode-field diameter of approximately 3.6 μm and fiber-tip 3D-printed microlens arrays for collimating and coherently combining the individual core outputs.^[13]^ The smaller and anisotropic channel spacings required here could be obtained using a customized core arrangement or an additional demagnifying relay. The spatially separated single-mode cores exhibit low inter-core crosstalk and small inter-core group-delay differences, which are favorable for coherent wavefront control. The relative inter-core phases may be measured interferometrically and compensated by channel-dependent phase offsets, while a polarizer after the fiber or a polarization-maintaining multicore fiber can provide a common polarization state.

The above schemes establish the physical feasibility of generating the required input scale. In addition, the inverse-design formulation provides flexibility for incorporating experimentally calibrated, nonideal input fields. In principle, the inverse-design formulation can accommodate experimentally characterized input fields that deviate from perfectly ideal Gaussian profiles. The relevant requirement here is that the input channels remain spatially distinguishable, with limited spatial overlap, so that separate input-to-output vector-mode mappings can be established. In a future experimental implementation, the calibrated complex vectorial field of each channel, including its amplitude, phase, polarization, spatial profile, and wavefront curvature, could be incorporated as the input boundary condition of the inverse-design model. The experimentally determined distance between the input plane and the first metasurface could likewise be included in the propagation operator. The VDNN could therefore be re-optimized for stable, experimentally calibrated input conditions rather than relying exclusively on ideal Gaussian fields.

Note S6. Simulation and design process of VDNN

Figure S4. Schematic diagram of the co-simulation process.

The input Gaussian optical field propagates through three layers of diffraction in the DNN to obtain the output OAM optical field. The loss function is obtained by comparing the output field with the target field. Then, the gradient value is calculated through error backpropagation. The parameters of the meta-atom array on the phase surface are updated. Subsequently, the atomic library of Si meta-atoms with height $H=1.2\mu m$, unit period of $600nm$, and wavelength of $1550nm$ is scanned and compared to find the meta-atoms with the closest parameters. The true phase parameters are obtained and used to update the DNN diffraction layers. Iterative updates are performed to acquire the final phase distribution.

Note S7. Analysis of efficiency loss mechanisms

Figure S5. Transmittance spectra of the x and y polarizations corresponding to the three-layer metasurface.

Figure S5 presents the transmittance spectrum of the database-matched meta-atoms in the three metasurface layers. The first and second rows correspond to the transmission coefficients under x- and y-polarized illumination, respectively. The arithmetic mean of the transmission coefficients over all selected meta-atoms is approximately 0.92. Because the database spectra are obtained for unrotated meta-atoms, a coordinate transformation is required to calculate the transmission response of each rotated meta-atom

$$u=E_{x}\cos\theta+E_{y}\sin\theta$$

$$v=E_{y}\cos\theta-E_{x}\sin\theta$$

where u and v represent the electric-field components projected onto the two principal axes of the rotated meta-atom and $\theta$is the in-plane rotation angle of the meta-atom, as shown in Figure 2 (a) in the main text. The local power transmittance of each meta-atom can therefore be calculated as:

$$T_{\text{eff}}(x,y)=\frac{t_{x}\left|u\right|^{2}+t_{y}\left|v\right|^{2}}{\left|E_{x}\right|^{2}+\left|E_{y}\right|^{2}}$$

First, to quantitatively isolate the efficiency loss caused by the nonideal transmission response of the meta-atoms, we need to analyze this contribution independently of finite-aperture truncation and additional full-wave effects. Taking the *P*_2,1_ input channel as an example, Fig. S6 shows the relationship between the normalized incident-field intensity and the local power transmittance of all 3600 meta-atoms in each layer. The horizontal axis represents the normalized local incident intensity, whereas the vertical axis represents the corresponding power transmittance of the matched meta-atom.

Figure S6. Field-weighted effective transmission without the finite-aperture truncation.

Because different meta-atoms receive different amounts of optical power, the arithmetic mean of their transmission coefficients does not accurately represent the effective transmission of the complete layer. We therefore define the intensity-weighted transmittance of the layer as:

$$T_{\text{layer}}=\frac{\sum_{x,y} I\left(x,y\right)T_{\text{eff}}\left(x,y\right)}{\sum_{x,y} I\left(x,y\right)}$$

According to Figure S6, intensity-weighted power transmittance of three layers under *P*_2,1_ input condition can be obtained as $T_{\text{layer 1}}=0.9287$,$T_{\text{layer 2}}=0.9209$and $T_{\text{layer 3}}=0.9061$, which are matched with arithmetic mean transmission. Therefore, the efficiency considering meta-atoms transmittance is around $\text{Eff}_{T}=77.49\%$.

In the second step, the influence of the finite aperture is considered in addition to the meta-atom transmission loss. The simulated output fields presented in Fig. 3 of the main text were obtained under ideal conditions, assuming an infinite transverse aperture and lossless transmission during the inter-layer transmission, the aperture truncation is only considered at the receiver plane, leading to an 88.02% efficiency. In the full-wave model, however, a rectangular aperture matching the physical size of the metasurface was introduced to represent its finite active area. Therefore, part of the diffracted field extending beyond the$36\text{μm}\times36\text{μm}$ apertures cannot be transmitted to the subsequent layer.

Figure S7. Intensity distributions at the output planes of the three metasurface layers.

Figure S7 presents the intensity distributions at the output planes of the three metasurface layers for the *P*_2,1_ input condition. The first row shows the intensity distributions calculated without aperture truncation, whereas the second row highlights the field components located outside the finite aperture. The fractions of optical power within and outside the aperture are also indicated. As the field propagates through successive layers, diffraction broadening causes an increasing fraction of the power to extend beyond the active metasurface area, resulting in progressively larger aperture-truncation loss. The calculated aperture transmittance after three layers is. After jointly considering the meta-atom transmission response and finite-aperture truncation, the overall power transmission is calculated as.

Figure S8. Stepwise quantitative analysis of efficiency degradation for different input channels. Steps 1–4 represent finite-aperture loss, meta-atom transmission loss, aperture and transmission combined effect, and the full-wave simulation result, respectively.

Finally, additional efficiency degradation occurs in the full-wave simulation of the cascaded metasurfaces. Because the meta-atom database is constructed under local periodicity approximation, which cannot fully capture the intercoupling effects. The resulting amplitude and phase deviations accumulate during propagation through the three metasurface layers, reducing the efficiency from approximately 68% after accounting for the transmission and finite-aperture losses to 55.75% in the full-wave simulation. The complete efficiency-decomposition results for different input channels are summarized in Figure S8. Similar degradation trends are observed for all channels, indicating that the residual efficiency reduction is primarily attributable to finite-aperture truncation, transmission loss and local periodicity approximation.

Note S8. Reasons for the slight decrease of skyrmion number

1. The algorithm did not effectively converge to the global optimum, resulting in a mismatch between the polarization distribution of the output optical field of the metasurface and the target field.
2. The sacrifice of transmission efficiency during the arrangement of the metasurface affects the intensity of the local optical field, thereby influencing the local polarization state distribution under the combined output conditions.
3. The parameter scanning strategy for the meta-atoms involves a “local periodic approximation” assumption, which neglects the intercoupling effects within the metasurface. This effect leads to a degradation in the quality of the output optical field.

Note S9. Free-Space Propagation of the Generated Optical Skyrmions

Optical skyrmions possess nontrivial topological vector-field textures and intrinsic robustness, offering potential as robust information carriers in optical communication and information encoding. Previous studies have demonstrated that the stable preservation of skyrmionic field configurations can support robust communication under structural perturbations and facilitate free-space information transport. ^[14,15]^ In particular, the propagation stability of the polarization texture is an important factor affecting their applicability to free-space communication. For some skyrmion-generation schemes based on high-NA focusing, the textures degrade rapidly away from the focal plane, limiting free-space information transport. ^[16]^

The VDNN proposed here can directly generate and multiplex micrometer-scale skyrmions without high-NA focusing. The post-target propagation property of Néel- and Bloch-type skyrmions over 10 μm is analyzed, including intensity distributions, polarization textures, normalized Stokes-vector fields, and skyrmion numbers. As shown in Figure S9, the characteristic Néel- and Bloch-type textures remain clearly identifiable throughout propagation, although moderate fluctuations in the skyrmion numbers are observed. These variations may mainly result from the nonideal generated skyrmion states and the sensitivity of the skyrmion-number calculation to low-intensity boundary fields. Further improvements could be achieved by introducing propagation-dependent topological constraints into the loss function and increasing the metasurface area. These results demonstrate the propagation stability of the generated textures and provide a basis for future VDNN-enabled skyrmion information encoding and optical communication.

Figure S9. Free-space propagation of representative Néel- and Bloch-type skyrmions generated by the VDNN. (a) The vectorial polarization distributions and topological Stokes-vector textures. (b) The evolution of the skyrmion numbers S over a propagation distance of 10 μm.

Note S10. Broadband performance of the six channels.

Figure S10 summarizes the wavelength-dependent coupling efficiency and OAM mode purity obtained from full-wave simulations. The Gaussian modes for *P*_2,1_ and *P*_2,2_ input conditions exhibit a broader operating bandwidth, whereas the higher-order OAM modes are more sensitive to wavelength detuning because of their more complex spatial-phase distributions. The maximum efficiency and mode purity occur near 1535 nm, rather than exactly at the design wavelength of 1550 nm. This spectral shift mainly arises because the meta-atom library was obtained under single-wavelength condition. Overall, the broadband behavior is jointly determined by wavelength-dependent interlayer free-space propagation and the dispersive response of the meta-atoms. Within 1550 ± 15 nm, both the coupling efficiency and OAM mode purity remain close to their peak values and exhibit only minor degradation.

Figure S10. Wavelength-dependent performance of the VDNN under full-wave simulations. (a) Coupling efficiency and (b) OAM mode purity of the six output channels over the wavelength ranges from 1460 to 1610 nm.

Note S11. The remaining five types of skyrmion formulas, topological textures, and generation operations involved

Table S2. The information on the remaining five types of skyrmions generated

| **Type** | **Expression** | **Texture** | **Operation** |  |
| --- | --- | --- | --- | --- |
| Anti | $\Psi=\psi_{0,0}\mathbf{R}+\psi_{1,-1}\mathbf{L}$ | Saddle$(-\frac{\pi}{4})$^*^ | $P_{3,2}$ and $P_{2,1}$;$\theta=\frac{\pi}{2}$; | |
| n.p. ^**^-Néel | $\Psi=\psi_{0,0}\mathbf{L}+\psi_{1,1}\mathbf{R}$ | Hedgehog | $P_{1,1}$ and $P_{2,2}$;$\theta=0$; | |
| n.p.-Bloch | $\Psi=\psi_{0,0}\mathbf{L}-i\psi_{1,1}\mathbf{R}$ | Spiral | $P_{1,1}$ and $P_{2,2}$;$\theta=0$; | |
| n.p.-Anti | $\Psi=\psi_{0,0}\mathbf{L}-i\psi_{1,-1}\mathbf{R}$ | Saddle | $P_{3,1}$ and $P_{2,2}$;$\theta=\frac{\pi}{2}$; | |
|  | $\Psi=\psi_{0,0}\mathbf{L}+\psi_{1,-1}\mathbf{R}$ | Saddle$(-\frac{\pi}{4})$ | $P_{3,1}$ and $P_{2,2}$;$\theta=\frac{\pi}{2}$; |  |

*Saddle$(-\frac{\pi}{4})$means that its orientation differs from the inverted saddle point in the text by $\frac{\pi}{4}$.

**n.p. refers to negative polarity.

Note S12. The remaining five types of bimeron formulas, topological textures, and generation operations involved

Table S3. The information on the remaining five types of bimerons generated

| **Type** | **Expression** | **Texture** | **Operation** |
| --- | --- | --- | --- |
| Anti | $\Psi=\psi_{0,0}\mathbf{R}+\psi_{1,-1}\mathbf{H}$ | Saddle$(-\frac{\pi}{4})$^*^ | $P_{3,1},P_{3,2},P_{2,1}$;$\theta=\frac{\pi}{2}$; |
| n.p. ^**^-Néel | $\Psi=\psi_{0,0}\mathbf{L}+\psi_{1,1}\mathbf{H}$ | Hedgehog | $P_{1,1},P_{1,2},P_{2,2}$;$\theta=0$; |
| n.p.-Bloch | $\Psi=\psi_{0,0}\mathbf{L}-i\psi_{1,1}\mathbf{H}$ | Spiral | $P_{1,1},P_{1,2},P_{2,2}$;$\theta=0$; |
| n.p.-Anti | $\Psi=\psi_{0,0}\mathbf{L}-i\psi_{1,-1}\mathbf{H}$ | Saddle | $P_{3,1},P_{3,2},P_{2,2}$;$\theta=\frac{\pi}{2}$; |
|  | $\Psi=\psi_{0,0}\mathbf{L}+\psi_{1,-1}\mathbf{H}$ | Saddle$(-\frac{\pi}{4})$ | $P_{3,1},P_{3,2},P_{2,2}$;$\theta=\frac{\pi}{2}$; |

* Saddle$(-\frac{\pi}{4})$means that its orientation differs from the inverted saddle point in the text by $\frac{\pi}{4}$.

**n.p. refers to negative polarity.

Note S13. Impact of transmittance spectrum on device performance

To investigate the impact of transmittance spectrum on device performance, the following section will employ both numerical simulation methods and full-wave finite-difference time-domain (FDTD) simulations using Lumerical FDTD to evaluate the system's performance. We have already shown output OAM mode distribution, polarization distribution and energy conversion efficiency in Figure 3. The output optical field patterns and polarization distributions of the two methods are very close to the ideal field. However, under numerical simulation conditions, the overall optical field conversion efficiency averages around 90%, while under full-wave FDTD simulation conditions, it decreases to approximately 55%. Therefore, the impact of meta-atom transmittance deviation is a global decrease in energy conversion efficiency. This is because the transmittance deviation is distributed relatively uniformly across the entire metasurface structure and can be regarded as pseudo-randomly distributed loss noise.

Figure S11. (a) Phase distribution given from the ideal OAM beams, numerical simulation results, and FDTD simulation outputs. (b) OAM purity crosstalk matrix from numerical simulation (without transmission loss) and FDTD simulation (with transmission loss).

In addition, for this work, phase modulation is as important as amplitude modulation of the optical field. Therefore, the phase modulation performance of the device under numerical simulation and FDTD simulation conditions will also be evaluated below. Figure S11 (a) shows the phase distribution results under the two methods. It can be seen that in the regions with higher intensity; the phase distribution matches well with ideal target distribution. However, for regions with lower intensity, the phase distribution has been distorted to some extent. This is because, in the loss function design adopted in this paper (see Equation 4 in main text), the loss function term that encourages phase matching is directly designed based on the cross-correlation function of the complex valued optical field. Therefore, for the phase distribution in regions with lower field intensity, the loss function value is smaller, resulting in a reduced ability to modulate the phase distribution. However, this result does not affect the performance of the device. At the same time, it can also be seen that the consistency of the optical field phase distribution under two methods is relatively high. Therefore, it can be concluded that the transmission rate error of the meta-atoms has a relatively small impact on the phase distribution. In the meta-atom arrangement strategy, it is feasible to sacrifice the transmission rate of some meta-atoms to ensure the matching of the phase distribution.

At the same time, it is necessary to evaluate the crosstalk performance. Here, the mode purity of the output OAM optical field is used as an indicator to characterize its crosstalk matrix shown in Figure S11 (b). It can be seen that the transmission loss has a relatively small impact on the OAM purity crosstalk matrix. This phenomenon is consistent with amplitude and phase distribution of the output optical field, so that the impact of transmission loss on the optical field mainly causes overall decrease in conversion efficiency, with limited influence on optical field quality. The coupling efficiency and OAM mode purity are calculated using the following equations. The efficiency Eff is defined as the ratio of the optical power within the designated output region to the total incident power, whereas the OAM mode purity *P*_OAM_ is defined as the normalized overlap between the output field and the target OAM mode.

$$\text{Eff=}\frac{\iint_{r} \left|E_{\text{out}}\right|^{2}dxdy}{\iint\left|E_{\text{in}}\right|^{2}dxdy},P_{OAM}=\frac{\left|\iint E_{\text{out}}\cdot E_{tar}^{*}dxdy\right|^{2}}{\iint\left|E_{\text{out}}\right|^{2}dxdy\cdot\iint\left|E_{\text{tar}}\right|^{2}dxdy}$$

The local vectorial phase residual between target phase distribution and simulation results can be obtained from Figure S11 (a), as shown in Figure S12. The phase quality within a selected region $\Omega$is evaluated using the mean absolute phase error and phase coherence factor,

where *N* is the number of sampling points in the region $\Omega$. A lower MAE indicates a smaller average phase deviation, while a higher *C* indicates a more concentrated phase-residual distribution. Therefore, good phase matching is characterized by both low MAE and high *C.*

Figure S12. Local vectorial phase residual between the target and simulated output fields for the six input channels.

Figure S13. Threshold-dependent phase quality in the high-intensity region. (a) Mean phase MAE and (b) phase coherence factor averaged over the six channels.

The output intensity and its peak value are defined as$I_{\text{peak}}$. For a normalized threshold $\eta$, the low- and high-intensity regions are defined as

$$\Omega_{\text{L}}\left(\eta\right)=\left\{r\left|I\left(r\right)\text{<}\eta I_{\text{peak}}\right.\right\},\Omega_{\text{H}}\left(\eta\right)=\left\{r\left|I\left(r\right)\geq\eta I_{\text{peak}}\right.\right\}.$$

As low-intensity pixels are progressively excluded, the phase MAE in $\Omega_{\text{H}}$decreases, while the phase coherence increases and gradually approaches saturation. At $\eta=5\%$, the averaged MAE and *C* in the high-intensity region are 0.128 rad and 0.983, respectively, as shown in Figure S13. Meanwhile, the low-intensity region contains only approximately 6.74% of the total optical energy. These results support the use of $I<5\%I_{\text{peak}}$as an empirical definition of the poorly controlled phase region.

Figure S14. Low-intensity regions defined by *I* < 5%*I*_peak_ for the six channels.

To directly evaluate its influence, uniformly distributed random phases were introduced into pixels satisfying $I<5\%I_{\text{peak}}$, as shown in Figure S14. The same random phase was applied to both polarization components in the common perturbation only in $\Omega_{\text{L}}$, while independent random phases were used in the independent perturbation. As shown in Figure S15, the variations in OAM mode purity remain on the order of 10^-4^ under both perturbation conditions, indicating that the relatively large phase errors in low-intensity regions produce only minor changes in the output modal characteristics.

**Figure S15. OAM mode purity perturbation for the six channels after randomizing the phase within the poorly -phase-control region with *I* < 5%*I*_peak_. Common perturbation applies the same random phase to both polarization components, whereas independent perturbation applies separate random phases to the two components.**

Note S14. Impact of alignment error on device performance

Alignment errors may occur during the actual assembly and adjustment of the three-layer metasurface. Therefore, it is necessary to evaluate the impact of alignment errors on the purity of OAM and the overall optical field conversion efficiency. The alignment errors can be categorized into the lateral and longitudinal displacement errors of the metasurface$(X_{offset},Y_{offset})$ and the layer spacing error $(D_{offset})$, as shown in Figure S16 (a). The unit for the lateral and longitudinal displacement errors can be the number of meta-atom unit pixels. The fundamental mode and the first-order OAM mode under full-wave simulation conditions can be selected as analysis cases. Figure S16 (b) shows the impact of the lateral and longitudinal displacement errors of the first layer of the metasurface on the optical field conversion efficiency under the incident position *P*_2,2_ and the impacts of the lateral and longitudinal displacement errors of the first, second, and third layers of the metasurface under the incident position *P*_1,2_. It can be seen that regardless of which layer of the metasurface has the displacement error, or whether it is the fundamental mode or the first-order OAM mode, the decrease in optical field conversion efficiency exhibits a similar attenuation pattern. Therefore, for the conversion efficiency of optical field, the lateral and longitudinal displacement alignment errors should be controlled within 2 pixels, that is, 1.2 μm.

However, the impact of lateral and longitudinal displacement errors on the purity of the output optical field modes exhibits a completely different pattern compared to the efficiency, as shown in Figure S16 (c). It can be seen that for the fundamental mode, the mode purity is less sensitive to lateral and longitudinal displacement errors, while the first-order OAM mode exhibits higher sensitivity. This is because, for the fundamental mode, the incident optical field and the output optical field have the same mode and are closer to each other spatially. However, for the first-order OAM mode, both the spatial position and mode distribution of the corresponding incident optical field undergo significant changes. Therefore, the sensitivity to lateral and longitudinal displacement errors is higher for the first-order OAM mode. Moreover, it can be seen that the impact of lateral and longitudinal displacement errors on mode purity is greater for the diffraction layers at later positions than for the earlier layers. This is because alignment errors in the incident optical field can be partially compensated by the diffraction layers to some extent. However, if the final diffraction layer has displacement errors, the optical field loses the opportunity for compensation. As a result, the mode purity drops sharply within a few pixels of error. Therefore, combining the above analysis, lateral and longitudinal displacement errors should focus on the later diffraction layers, and the errors should be controlled within two pixels as much as possible.

**Figure S16. (a) Schematic definition of the lateral X-offset, Y-offset, and interlayer-spacing D-offset errors. (b) Effects of lateral misalignment on conversion efficiency. (c) Effects of lateral misalignment on OAM mode purity. (d) Effects of interlayer-spacing error on conversion efficiency and OAM mode purity.**

As a diffractive neural network, the spacing between diffractive layers is also an important structural parameter of the network. The size of the layer spacing determines the irradiation range of the preceding optical field on the subsequent metasurface structure. Therefore, it is necessary to evaluate the impact of layer spacing errors on the purity of OAM, overall optical field conversion efficiency, as shown in Figure S16 (d). The upper two panels demonstrate the impact of the layer spacing error $D_{offset}$ on optical field conversion efficiency under incident positions *P*_2,2_ and *P*_1,2_. Analogously, the lower two panels show the impact on optical field mode purity. Here, the impact of layer spacing errors on device performance is assessed in units of wavelength, with the error analysis focusing on the range $D_{offset}\in[-5\lambda,5\lambda]$. It can be seen from the figure that the fundamental mode optical field remains more robust against layer spacing errors in terms of both optical field conversion efficiency and mode purity compared to the first-order OAM mode, which exhibits higher sensitivity to layer spacing errors. Overall, if the layer spacing error is controlled within the range $[-2\lambda,2\lambda]$, its impact on OAM mode purity and overall optical field conversion efficiency is relatively small. It can be concluded that the impact of layer spacing errors on device performance is much smaller than that of lateral and longitudinal alignment errors. Therefore, in subsequent practical experiments, lateral and longitudinal errors should be the primary focus.

To assess the tolerance of the proposed VDNN to rotational misalignment, an in-plane angular offset was introduced between the optical field emerging from the second layer and the third metasurface. The relative rotation angle was varied from -10° to 10° with an interval of 1°, and the corresponding output fields were recalculated for all six input channels. Figures S17 (a) and (b) show the coupling efficiency and OAM mode purity, respectively, as functions of the rotational misalignment. Representative output intensity distributions for three selected channels are also provided in Figures S17 (c) to illustrate the evolution of the output fields.

The fundamental-mode channels *P*_2,1_ and *P*_2,2_ exhibit the highest tolerance in both metrics. For the higher-order OAM channels, the coupling efficiencies display broadly similar degradation trends, whereas the mode-purity responses are distinctly channel dependent. In particular, *P*_1,1_ and *P*_3,1_ retain higher OAM purities than *P*_1,2_ and *P*_3,2_ under increasing rotational offsets. Consistent with this trend, the output field of *P*_1,2_ exhibits more pronounced modal distortion at large misalignment angles, as can be seen in Figures S17(c). This asymmetric tolerance may arise from the common 45° linear polarization of the input beams, which introduces polarization-dependent spatial asymmetry into the input-field distribution and consequently leads to unequal rotational sensitivities among symmetrically positioned channels.

**Figure S17. Rotational-misalignment tolerance of the proposed VDNN. (a) Coupling efficiency and (b) OAM mode purity of the six output channels as functions of the relative in-plane rotation between the optical field emerging from the second layer and the third metasurface. (c) Output intensity distributions under different rotational offsets.**

Overall, the VDNN maintains relatively stable coupling efficiency and OAM mode purity within a rotational-misalignment range of approximately ±4°. Beyond this range, the accumulated mismatch between the propagated field and the phase–polarization modulation of the third metasurface results in increasingly evident efficiency reduction and modal degradation.

Reference

1. F. Y. Yue, D. D. Wen, J. T. Xin, B. D. Geradot, J. Li and X. Z. Chen, “Vector vortex beam generation with a single plasmonic metasurface,” *ACS Photonics*, vol. 3, no. 9, pp. 1558-1563, 2016.
2. Y. J. Shen, E. C. Martinez, and C. Rosales-Guzmán, “Generation of Optical Skyrmions with Tunable Topological Textures,” *ACS Photonics*, vol. 9, no. 1, pp. 296-303, 2022.
3. K. Singh, P. Ornelas, A. Dudley, and A. Forbes, “Synthetic spin dynamics with Bessel-Gaussian optical skyrmions,” *Opt. Express*, vol. 31, no. 10, pp. 15289-15300, 2023.
4. H. A. Teng, J. Z. Zhong, J. Chen, and Q. W. Zhan, “Physical conversion and superposition of optical skyrmion topologies,” *Photon. Res.*, vol. 11, no. 12, pp. 2042-2053, 2023.
5. J. Wang, J. Chen, F. Yu, R. Chen, J. Wang, Z. Zhao, W. Lu, “Unlocking ultra-high holographic information capacity through nonorthogonal polarization multiplexing,” *Nat Commun* *15*(1), 6284, 2024.
6. G. Soma, K. Komatsu, Y. Nakano, “Complete vectorial optical mode converter using multi-layer metasurface,” *Nat Commun* 16, 7744, 2025.
7. W. M. Zhen, Z. M. Qing, W. X. Yan, et al., “Reconfiguring optical skyrmion topology in free space,” *Optica*, vol. 13, no. 2, pp. 188-194, 2026.
8. H. Hu, G. Wang, L. Wang, S. Zhao, “Multi-mode vector vortex beams generation enabled by a rotatable D2NN,” *Optics Express*, *34*(1), 743-753, 2026.
9. J. Xiao, G. Wang, L. Wang, S. Zhao, “D2NN-enabled generation of Poincaré sphere vector vortex beams with light-controlled polarization and topological charge,” *Physica Scripta*, *101*(21), 215514, 2026.
10. K. Nishimura, H. Sakai, T. Ando, T. Tomita, S. de Léséleuc, “Optimization-based hologram design for fine optical tweezer arrays and extension of super-resolution criteria,” *Physical Review A*, *113*(1), 013119, 2026.
11. D. Bowman, T. L. Harte, V. Chardonnet, C. De Groot, S. J. Denny, G. Le Goc, G. D. Bruce, “High-fidelity phase and amplitude control of phase-only computer-generated holograms using conjugate gradient minimisation,” *Optics express*, *25*(10), 11692-11700, 2017.
12. M. Prossotowicz, D. Flamm, A. Heimes, F. Jansen, H. J. Otto, A. Budnicki, U. Morgner, “Dynamic focus shaping with mixed-aperture coherent beam combining,” *Optics Letters*, *46*(7), 1660-1663, 2021.
13. S. Sivankutty, A. Bertoncini, V. Tsvirkun, N. Gajendra Kumar, G. Brévalle, G. Bouwmans, H. Rigneault, “Miniature 120-beam coherent combiner with 3D-printed optics for multicore fiber-based endoscopy,” *Optics Letters*, *46*(19), 4968-4971, 2021.
14. Y. Jian, Y. Wang, H. Li, et al. “Non-Hermitian Skyrmion for All-In-One Devices: Wireless Power Transfer, Sensing, and Communication.” *Laser & Photonics Reviews* 20, no. 13: e02662. 2026.
15. Y. Liu, L. Zhu, X. Xie, “Broadband coloured skyrmions generated by on-chip ferroelectric spherulites.” *eLight* 6, 18, 2026.
16. J. He, C. Song, W. Li, F. Sun, and G. Yuan, “Subdiffraction Confinement and Non-Diffractive Propagation of Optical Stokes Skyrmions Enabled by a Super-oscillatory Metalens.” *Laser & Photonics Reviews* e03051, 2026.
